# Predicting the impact of urban flooding using open data

**DOI:** 10.1098/rsos.160013

**Published:** 2016-05-25

**Authors:** Nataliya Tkachenko, Rob Procter, Stephen Jarvis

**Affiliations:** 1 Warwick Institute for the Science of Cities, University of Warwick, Coventry CV4 7AL, UK; 2 Department of Computer Science, University of Warwick, Coventry CV4 7AL, UK

**Keywords:** Web search, Google Analytics, flood risk management, urban resilience, predictive analytics

## Abstract

This paper aims to explore whether there is a relationship between search patterns for flood risk information on the Web and how badly localities have been affected by flood events. We hypothesize that localities where people stay more actively informed about potential flooding experience less negative impact than localities where people make less effort to be informed. Being informed, of course, does not hold the waters back; however, it may stimulate (or serve as an indicator of) such resilient behaviours as timely use of sandbags, relocation of possessions from basements to upper floors and/or temporary evacuation from flooded homes to alternative accommodation. We make use of open data to test this relationship empirically. Our results demonstrate that although aggregated Web search reflects average rainfall patterns, its eigenvectors predominantly consist of locations with similar flood impacts during 2014–2015. These results are also consistent with statistically significant correlations of Web search eigenvectors with flood warning and incident reporting datasets.

## 1. Introduction

The biggest issue for flood risk management in urban areas is the prediction that under climate change there will be considerably more flooding in these areas. Specifically, in the UK, flooding is considered to be one of the biggest problems that the country is facing today, with climate projections suggesting that the increase in total rainfall will provoke major, more frequent and less predictable flood events [1]. The impact of floods on housing is also increasing due to the ongoing development of settlements in flood-prone areas, together with the rising vulnerability of assets to risk [2,3].

The phenomenon of flooding is extremely complex and subject to change. Incidents are no longer restricted to obvious areas where a river or stream exists; many urban floods are simply caused by huge amounts of rain falling very quickly (flash floods) in an area where the drainage system is unable to cope or due to unexpected underground basin recharge and rise of the groundwater levels [4]. As a consequence, there is an emerging motivation to understand how accurate our knowledge can be about flood risk—its location, timing and duration—and how data collection and analysis can assist us.

People usually make an effort to stay aware of what happens in their neighbourhoods, especially if their health, wellbeing or prosperity is at stake. It has already been shown that public information sufficiency, risk perception and self-efficacy can predict risk-information seeking behaviour [5–7].

As a particular type of information seeking, Web search has become a key reference source [8]. Although not always 100% accurate, it is fast and free of charge. Built-in traffic monitoring engines within websites help to collect data about information-seeking behaviour and one such example is Google Analytics, which is capable of tracking hourly information about unique page visits, number and duration of individual sessions at the level of cities and towns and—in some cases—at the level of postcodes. Google Analytics has found wide applications in business and website optimization [9–11], and is increasingly becoming one of the main generators of ‘big data’ records, alongside other systems that record, for example, our communications, travel and retail activities. A number of recent studies have provided evidence [12–16] that ‘big data’ can reveal a great deal about people's real-world, collective decision-making and responses to events and can even help to predict such phenomena, e.g. Hurricane Sandy [17].

To optimize the design of its Web-based services, the UK Environment Agency has also installed Google Analytics on its live flood warning pages. Interest has therefore emerged in analysing records of flood warning information seeking, which, coupled with geolocation records, could be potentially useful not only for Web designers, but also for flood risk modellers. In this study, we analyse whether Web-based information seeking about flood risk can help us understand how badly those locations have been, or may be, affected.

## 2. Data

**Figure 1.**
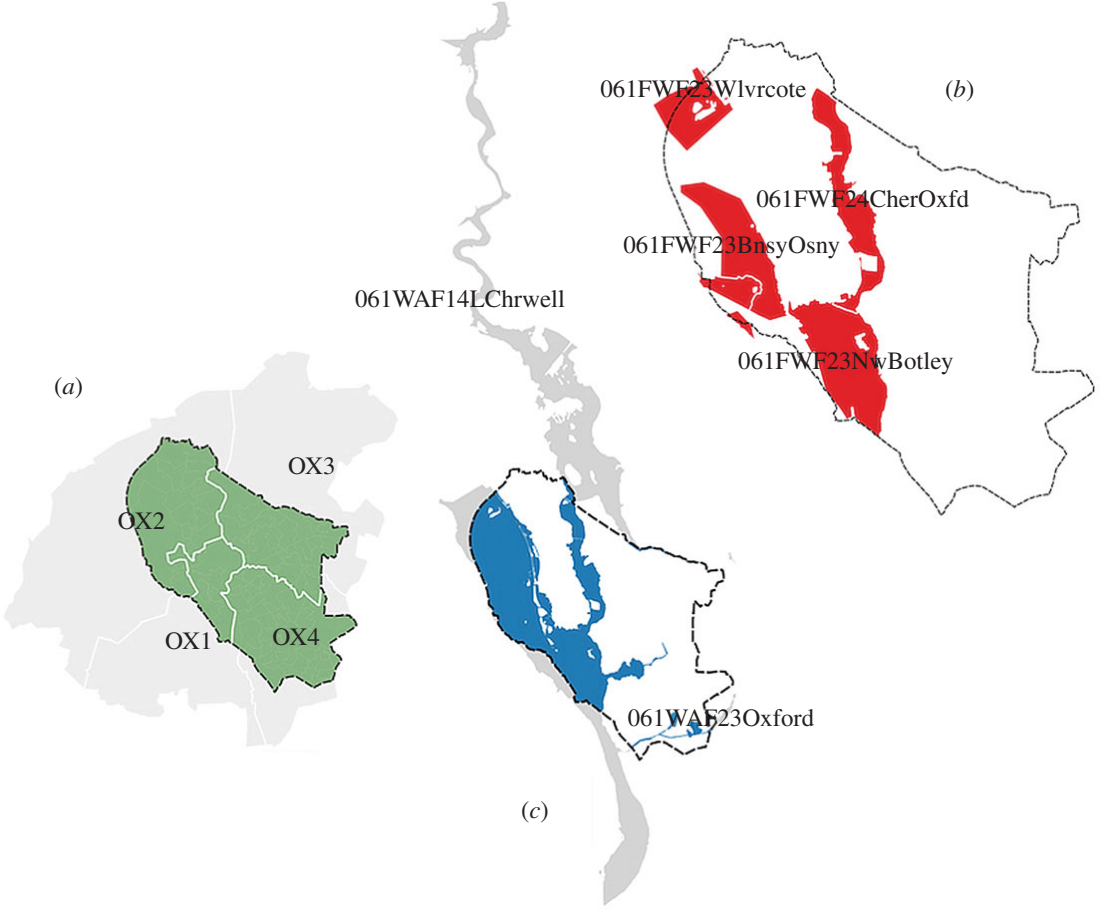
Examples of adjustments of the input datasets to city boundaries. (*a*) Floodline incident dataset is supplied for open use at postcode district level. All four postcode districts intersecting Oxford city boundaries are considered as ‘Oxford’ in our analysis. (*b*) Geographical extent of Historic Flood Warnings fragments close to the city boundaries. Oxford comprises four official flood-warning areas; each has a unique attributed ID and dates of the previously issued communications. (*c*) The attribute format of the Historic Flood Alerts dataset is very similar to the warnings, but has coarser spatial resolution: thus, Oxford city is covered by two such designations, which extend far beyond its boundaries. In our analysis, all the alerts and warnings issued in any of these fragments, if intersecting any part of the city, are considered attributable to that particular urban locality.

All datasets used in our analysis fall into the category of open data and are available free of charge. They can be divided into three main groups: (i) datasets available for direct download online, (ii) datasets available for public use, but where prior registration or permission of the data officer is required, and (iii) datasets contained in commercial databases, but accessible via application program interfaces or by crawling Internet resources. A complete list of the datasets used in this study is available in table 1. Owing to the varying geographies of the original data sources, some data pre-processing was necessary in order to standardize the analysis to city scales (figure 1).

**Table 1.** List of datasets.

dataset	source	geography	access
1. Google Analytics	Environment Agency	city/town	upon request
2. Historic Flood Warnings	Environment Agency	river/stream	direct download
3. Historic Flood Alerts	Environment Agency	river/stream	direct download
4. Incident Hotline Records	Environment Agency	postcode district	upon request
5. Rainfall Rank Ordered Statistics	Met Office	national/regional	direct download
6. UKCP09 Rainfall Grids	Met Office	5 × 5 km	upon request
7. Hazard Media Mentions	collected	city/town	electronic supplementary material

### 2.1. Environment Agency data

#### 2.1.1. Google Analytics records

In order to keep the public informed about risks, the Environment Agency maintains several interactive Web-based services, which provide both regionally summarized (‘Flood Warning Summary’, http://apps.environment-agency.gov.uk/flood/31618.aspx) and localized (‘Live Flood Warning Map’, https://flood-warning-information.service.gov.uk/) information about current flood risks. We requested Google Analytics metrics for views of these webpages for the period April 2014–March 2015. This period was marked by the effects of two trans-Atlantic hurricanes (‘Bertha’, 1–14 August 2014; and ‘Gonzalo’, 12–20 October 2014), and increased precipitation levels across the whole of the UK in January 2015 (figure 4). The dataset comprised recorded activity in more than 500 UK cities and towns over 1 year.

#### 2.1.2. Historic flood warning records

Summaries of historic flood warnings and alerts are available for free download from the Environment Agency Geostore website at: http://www.geostore.com/environment-agency/. The dataset employed in our analysis contained more than 2500 entries over the period from April 2014 to March 2015 across England and Wales. Messages classified as ‘alerts’ and ‘warnings’ correspond to either ‘areas that are at risk from low impact flooding’ or ‘discrete communities at risk of flooding’, respectively.

#### 2.1.3. Incidents hotline records

Incident hotline records are another Environment Agency dataset that is available upon request. The hotline (0800-80-70-60) is a service that has been put in place to enable community reporting on various environmental incidents of both anthropogenic and natural origins, including pollution to water or land, illegal fishing, illegal dumping of hazardous waste, erosion of watercourse banks and cases of surface water flooding to properties. Although by cases of surface water flooding, environmental institutions mean primarily flooding from nearby watercourses and pluvial ‘flash’ floods, it has been widely acknowledged in research literature [18–20] that the general public rarely differentiate between groundwater and surface water floods, so some degree of bias was expected in the dataset due to some localized cases of post-rainfall rise of the groundwater table.

Both the Incidents Hotline Records and the Google Analytics Records were supplied for research purposes. In accordance with the Data Protection Act 1998 and the Privacy and Electronic Communications Regulations 2003, Google Analytics Records were supplied at the city level, whereas the Incidents Hotline Records were supplied at the level of postcode districts (first four alphanumeric characters, e.g. ‘WS13’).

### 2.2. Met Office data

We used UK climate summaries as background information for flood-related information-seeking behaviour on the Web. (i) Rank ordered statistics for rainfall (mm) average across England and Wales, which is available for download from http://www.metoffice.gov.uk/climate/uk/summaries/datasets. These area data series date back to 1910, with allowances made to account for topographic, coastal and urban effects, where relationships are found to exist. (ii) UKCP09 grid text files of monthly rainfall values, which provide a matrix covering the whole of the UK. Each value represents an estimate for the centre point of 5 × 5** **km grid cells, which are identified using the Ordnance Survey National Grid, extended to cover Northern Ireland. The format of the grid text files allowed their import and manipulation in open source QGIS software. This dataset required both registration on the Met Office website and the specific time span needed for analysis.

### 2.3. Media mentions data

The degree of flood impact against which search activity is benchmarked in our study is represented by media coverage of flooding in cities, recorded by Google Analytics, during the time period April 2014–March 2015. Several authors previously highlighted that news media coverage tends to prioritize locations where people have been affected [21–23]. We therefore collected reports in various online news sources (see the electronic supplementary material) of each city PC1–4|*α* ≥ 0.5 (see ‘Material and methods’). For the purpose of our analysis, locations that appeared in the media were coded as ‘1’ and those that did not were coded as ‘0’ (see the electronic supplementary material).

## 3. Material and methods

**Figure 2.**
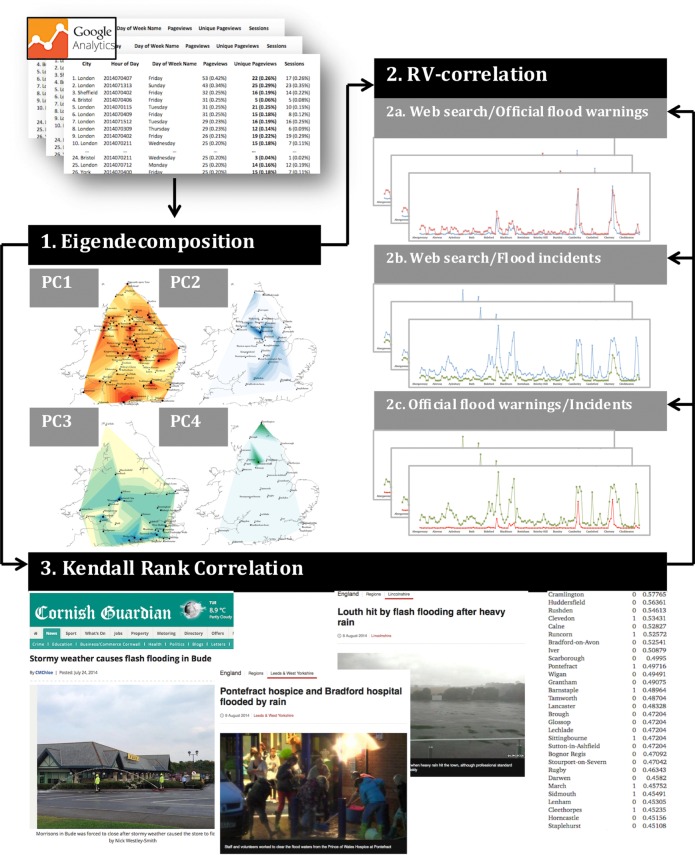
Method workflow.

**Figure 3.**
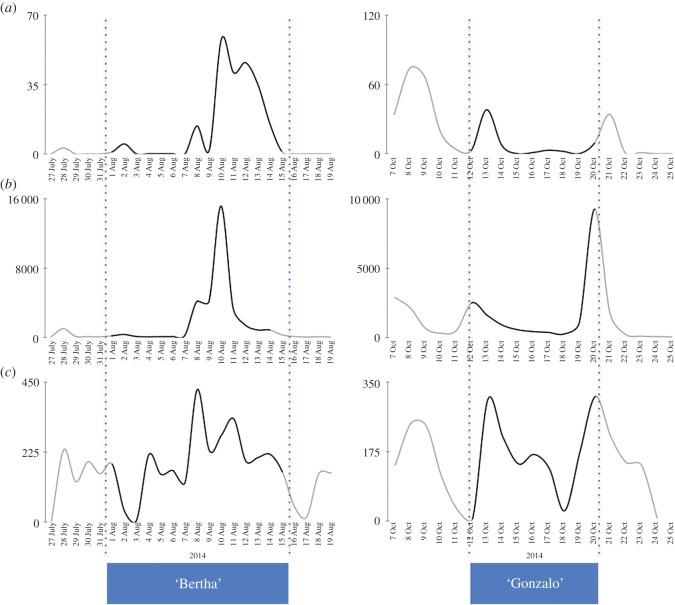
Temporal profiles of institutional and crowd activity during hurricanes ‘Bertha’ (1–14 August 2014) and ‘Gonzalo’ (12–20 October 2014). In total, a ±5-day buffer has been added to both events. (*a*) Flood warnings and alerts, issued by the Environment Agency, (*b*) Google Analytics of the traffic on the Environment Agency Live Flood Warning webpages, (*c*) floodline incidents reporting rates before, during and after both events.

We have developed a three-step analysis (figure 2) aimed at testing whether earlier or later engagement with flood risk information on the Web is correlated with actual hazard outcomes in cities with similar Web search behaviour patterns. Our analysis covers continuous datasets' entries from April 2014 to March 2015, and includes several major meteorological events, such as Hurricanes ‘Bertha’ (August 2014) and ‘Gonzalo’ (October 2014). Figure 3 demonstrates that it is quite difficult to define discrete boundaries within behavioural datasets owing to particular weather events.

First, we analysed spatio-temporal patterns in the annual Google Analytics dataset, in order to connect information needs to the geography of human activity on the Web. Similar to studies performed by Calabrese *et al*. [24], we adapted an eigendecomposition technique drawn from electronics and remote sensing to extract the discriminant features from our time-series data. We represented Web search activity in a particular city over time as a vector, and assembled the records from all (500+) cities and towns into a single covariance matrix. The procedure of eigendecomposition, which is often referred to as principal component analysis or nonlinear dimensionality reduction applied to time-series data, is a well-recognized and understood pattern recognition technique [25]. Decomposed in this way, the data allow us to subsequently determine the basic dimensions of the relationships between, for example, information-seeking activity in a number of cities and other factors, such as rainfall patterns, official flood warnings and rates of flood-related incidents in those locations.

**Figure 4.**
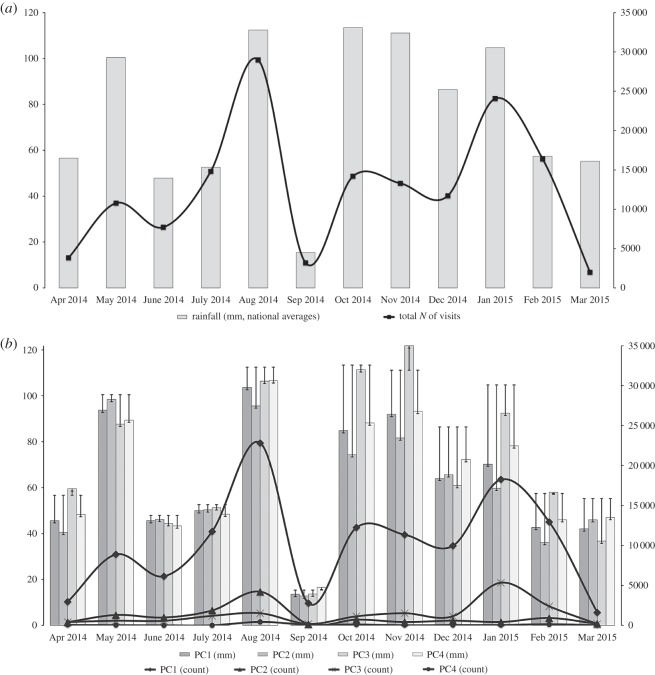
Relationships between monthly precipitation rates and information-seeking activity on the Web: (*a*) across England and Wales, (*b*) between Web activities in the locations, constituting PC1–4, and average rainfall amounts across those areas.

We performed eigendecomposition, which yielded around 12 eigenvector and coefficient pairs (table 2) and which have been ranked according to their values. In a similar manner to Calabrese *et al*. [24], we applied the mean squared error (MSE) test, which demonstrated that only the first four pairs were required to lower the error to below 0.1. Figure 4*b* illustrates four eigenvectors (PC1–4), which capture the decomposed information-seeking activity on the Web as compared to the corresponding average precipitation levels in the group of cities constituting each eigenvector; these we name *eigencities*, a similar term to *eigenplaces*, introduced by Calabrese *et al*. For the subsequent analysis, from each of the first four eigenvectors, we select only locations with the highest coefficient values greater than or equal to 0.5. The structure of the dataset is therefore: *N*_PC1_ = 96, *N*_PC2_ = 37, *N*_PC3_ = 41 and *N*_PC4_ = 30.

**Table 2.** Results of eigendecomposition.

	eigenvalue	variance	cumulative variance, %
Dim.1 (PC1)	7.471419 × 10	1.479489 × 10	14.79489
Dim.2 (PC2)	6.609348 × 10	1.308782 × 10	27.88271
Dim.3 (PC3)	5.791740 × 10	1.146879 × 10	39.35150
Dim.4 (PC4)	5.004381 × 10	9.909666	49.26116
Dim.5 (PC5)	4.932296 × 10	9.766923	59.02809
Dim.6 (PC6)	4.650346 × 10	9.208606	68.23669
Dim.7 (PC7)	3.910624 × 10	7.743811	75.98050
Dim.8 (PC8)	3.618921 × 10	7.166180	83.14668
Dim.9 (PC9)	3.141302 × 10	6.220399	89.36708
Dim.10 (PC10)	2.939832 × 10	5.821450	95.18853
Dim.11 (PC11)	2.429791 × 10	4.811468	100.00000
Dim.12 (PC12)	4.895469 × 10^−29^	9.693999 × 10^−30^	100.00000

The number of eigenvectors needed to reconstruct the original dataset with an acceptable error margin (MSE ± 0.1) usually depends on its complexity: the more chaotic the original dataset, the more information is needed to reconstruct it. In order to revert to the original, we need to multiply each component vector by its coefficient and sum the results

3.1 $${\text{S}}_{\mathrm{cities}}={\text{C}}_{1}{\text{V}}_{1}+{\text{C}}_{2}{\text{V}}_{2}+\cdots +{\text{C}}_{\text{n}}{\text{V}}_{\text{n}},$$

where ***S***_cities_ is the information-seeking behavioural profile for all cities present in the Google Analytics dataset, ***C***_*n*_ is the coefficient and ***V***_*n*_ is the eigenvector.

The decomposition of the information-seeking behaviour of 507 UK cities and towns into *eigencities* was performed on a 507 × 365 correlation matrix, which was derived as follows:

3.1*a* $${\text{C}}_{\mathrm{cities}}=\overset{365}{\underset{\mathrm{day}=1}{\sum }}{\text{E}}_{\mathrm{normalized}}(\lambda ,\delta ){\text{E}}_{\mathrm{normalized}}^{T}(\lambda ,\delta ),$$

where

3.1*b* $${\text{E}}_{\mathrm{normalized}}(\lambda ,\delta )=\frac{{\text{E}}_{\ln}(\lambda ,\delta )}{\underset{\lambda \in {\text{L}}_{507}}{\mathrm{mean}}[{\text{E}}_{\ln}(\lambda ,\delta )]}.$$

Next, we visualized relationships between official flood risk information issued to the public, reported incidents due to surface water flooding to properties and flood-related information seeking on the Web. This analysis sets up a framework, with which we can determine whether engagement with flood risk information occurred at an earlier (higher correlation with flood warnings and alerts) or later (correlation with incidents due to surface water flooding) stage of hazard development. For this purpose, we constructed three data matrices and employed paired RV (matrix correlation) analysis, which is a simple generalization of the squared Pearson correlation applied to multivariate data:

3.2 $$\mathrm{RV}(\text{X},\text{Y})=\frac{\mathrm{COVV}(\text{X},\text{Y})}{\sqrt{\mathrm{VAV}(\text{X})\mathrm{VAV}(\text{Y})}},$$

where $\mathrm{COVV}(\text{X},\text{Y})=\mathrm{Tr}(\Sigma_{\text{XY}}\Sigma_{\text{YX}}),\Sigma_{\text{XY}}=\text{E}({\text{X}}^{T}\text{Y})$ and $\mathrm{VAV}(\text{X})=\mathrm{Tr}(\Sigma_{\text{XX}}^{2}).$ Finally, we used Kendall's *τ* non-parametric measure of correlation between four pairs of two ranked variables: each of PC1–4 and corresponding binary values, which encode whether that locality had been recorded as affected or severely affected by flooding during the study period April 2014–March 2015:

3.3 $$\tau =\frac{\text{C}-\text{D}}{\text{C}+\text{D}}=\frac{\text{C}-\text{D}}{\text{n}(\text{n}-1)/2}=\frac{\text{C}-\text{D}}{\left(n/2\right)}=\frac{\text{C}-\text{D}}{\text{n}!/2!(\text{n}-2)!},$$

where ***C*** is concordant pairs and ***D*** is discordant pairs.

## 4. Results

Table 3 illustrates the combined presentation of the RV coefficients, depicting the similarity between information seeking matrices, official flood risk warnings and reported incidents due to flooding, and Kendall's *τ* coefficients, demonstrating ranked correlation between *eigencities* and either the presence (coded as ‘1’) or the absence (coded as ‘0’) of city/town coverage in the media due to flood events, over the period April 2014–March 2015. The strength of the information-seeking behaviour across all four components with either flood warnings or incident reporting indicates early or late public engagement, respectively. Kendall's coefficients demonstrate links between public engagement at either an early or a late stage of the flood event and the actual occurrence of the hazard, which as an impact benchmark dataset was preferable over probabilistic warning messages.

**Table 3.** Composite results.

	*N*|*α* ≥ 0.5	warnings	reporting	W : R	*τ*
PC1	96	0.372*	0.533**	0.211 n.s.	0.283***
PC2	37	0.187 n.s.	0.317*	0.401*	0.054 n.s.
PC3	41	0.643**	0.796**	0.550**	(−0.266)**
PC4	30	0.408*	0.191 n.s.	0.195 n.s.	(−0.044) n.s.

*p < 0.1, **p < 0.05, ***p < 0.001.

Results, extracted for the first four principal components, account for 50% of the total data variability, demonstrating that the most statistically significant Kendall coefficients (*τ *= 0.283*** and (−0.266)**) are for PC1 and PC3, respectively. Positive and negative values of those two components suggest that eigendecomposition is capable of differentiating between affected and unaffected (or, rather, between more affected and less affected) locations by analysing only Web search behaviour.

We extended the analysis of our initial findings to look at how they relate to the wider relationships between decomposed flood-related information seeking (PC1–4) and the formal flood risk-management infrastructure, i.e. warning and reporting in those locations. When we compared correlations of PC1 and PC3 with flood warnings and between warnings and reporting, respectively, we noticed the link between more active engagement with flood risk information during both warning and reporting stages of floods (RV(warnings)|PC3* *= 0.643** and RV(reporting)|PC3* *= 0.796**) and reduced hazard outcome in those locations: PC3| *τ *= (−0.266)**. Similarly, stronger and more statistically significant correlation of PC1 with incident reporting data (RV(reporting)|PC1* *= 0.533**) when compared with the warnings (RV(warnings)|PC1* *= 0.372*) suggests later engagement with risk warning information and worse hazard outcome in those locations: PC1|*τ *= 0.283***.

Although statistically insignificant, Kendall's coefficients for PC2 and PC4 are also consistent with the above-mentioned conclusions.

## 5. Discussion

In this study, we investigated whether there are links between flood risk-information seeking, which can be seen as a proactive behaviour vis-à-vis developing natural hazards, and actual outcomes in urban localities during the period from April 2014 to March 2015. We found that early engagement with flood risk information correlates with less severe hazard outcomes in the set of locations with similar search behaviour patterns, which are defined here as *eigencities*. Although total search volumes globally correspond to national average rainfall patterns (figure 4), it can be argued that different principal components are responses, not necessarily linear, to rainfall patterns at different spatial scales: from national to regional and to the most local. In such instances, principal components, or *eigencities*, represent a surrogate measure of the relationship between actual natural phenomena (e.g. precipitation levels) and local knowledge/habits/preferences of how to deal with the prospective or ongoing flood event.

The significance of this study can be illustrated by its numerous implications for social and environmental sciences, touching on both their theoretic and empirical aspects. First, the results align with several previous studies [5,26], and especially with protection motivation theory (PMT) [27,28], according to which threat and coping appraisals (or ‘information seeking’) correlate positively with protective responses. Protective responses are defined here as ‘those that prevent monetary or physical damage if an event actually occurs, and are taken if the threat appraisal and the coping appraisal are high’, and are in contrast to non-protective responses, such as denial or unfounded optimism. According to Grothmann & Reusswig [29], self-protective behaviour by residents of flood-prone urban areas can reduce monetary flood damage by 80% and reduce the need for public risk management. However, the literature in the field of psychology also indicates that if residents at risk rely on the efficacy of public or administrative flood protection, they take less precautionary action themselves; there is therefore scope for future studies to look into combined action of private damage prevention by households and levees built by public agencies to prevent floodwaters reaching people's doorsteps. In addition, despite the general alignment of our results with PMT and similar studies, other literature [30,31] also points to a close relationship between flood mitigation undertakings and flood risks, property values, government payrolls and population densities. There is therefore a strong argument for further study that uses these additional variables, in order to examine in more depth relationships between information seeking and motivation to act in the face of a hazard.

Information seeking is also seen as a novel component in the next generation of environmental models [32,33]. Increasingly, natural hazard risks and environmental amenities are becoming part of empirical agent-based land market modelling [34], where particular attention is now being paid to capturing the impact of changes in individual risk perceptions on land use choices. And while statistical relationships are established between risk perception and information seeking [5], there is a growing interest in understanding how quantifiable the links are between information-seeking activities and personal uptake of flood resilient measures.

Another set of agent-based models look into dynamic logistical systems of human and technological interaction during flood events [35,36]. They are largely built on topography, buildings and road networks, but lack information on large-scale social behaviour data. These multi-agent simulations are usually coupled with hydrodynamic models to estimate the vulnerability of individuals to flooding under different storm conditions; however, they could greatly benefit from data where individuals are treated as less passive agents [37,38].

Normative decision-making models are the most common tools for the estimation of economic benefits of meteorological services [39]. Our study suggests ways forward for next-generation normative valuation of meteorological services, where technology adopters and non-adopters can be identified using digital behavioural datasets, and where realistic input is a strong requirement for the prescriptive model.

As previously mentioned, eigendecomposition of information seeking on the Web could assist in the evaluation of various environmental policy instruments, where proactive communities play a key role. For example, implementation of flood resilient technologies (FReTs) at the individual property level provides a previously untapped resource to reduce flood damage to buildings [3]. The assessment of FReTs at the individual building level is gaining greater importance, in part because their targeted uptake is often constrained by a lack of informed knowledge about their performance. While the benefits of FReTs are defined as direct tangible damage avoidance to properties at risk, urban flood barrier systems, which are installed some distance from groups of buildings, also protect specific locations up to a certain threshold. Coupled with the results presented in this study, accessible information on flood barrier location, type and age, can help design a probabilistic decision-support tool to target more balanced FReTs across various urban scales.

Information seeking, sharing and implementation of resilience measures can be regarded as a particular type of risk-management behaviour, but which is not captured by current flood communication systems. Tracking the way people behave but also make changes to their properties and surrounding environment can open new directions for behavioural research, specifically, for environmental habit tracking and re-calculation of risks in prediction systems that are currently based solely on demographic and economic variables [37,40–42]. While this study has limitations, one of which is the use of a single information-seeking dataset (we do not employ Facebook groups and Twitter, for example), it does provide invaluable insights into complex relationships between flood risk information seeking on the Web and real-world, public behaviour in conditions of varying and extreme weather events.

## Supplementary Material

Method description and data entries for analysis in file 'RSOS160013_supplementary_material

## Data accessibility

All datasets used in this study are available for free either via direct download or upon request (see ‘Data’) as open data from the Environment Agency and Met Office. Methods for extraction and the records of cities' mentions in the media due to flood events are listed in the electronic supplementary material.
