# Supplementary material for: Predicting the impact of urban flooding using open data
Source: R Soc Open Sci. 2016 May 25;3(5):160013. doi: 10.1098/rsos.160013 (PMC4892441; doi:10.1098/rsos.160013)
Supplement: Method description and data entries for analysis in file 'RSOS160013_supplementary_material [file rsos160013supp1.pdf]

ROYAL SOCIETY
OPEN SCIENCE

R. Soc. open sci.
doi:10.1098/not yet assigned

Supplementary material

Extracting Hazard Media Mentions from the Web

The collection of media mentions of whether particular events took place in particular locations can be extracted either from commercial databases, such as Factiva© (business and research information tool from Dow Jones & Co., accessible by subscribed users and organisations from http://www.dowjones.com/products/product-factiva/) or with by using data extraction techniques in the Google Search engine. We used the later method.

Because Google is generally restrictive about access provision to its search results, several methods could be utilised to extract the sample of the URLs of interest. The complexity of the method can depend on several factors, specifically, on the level of the researcher's technical expertise as both manual and automated methods are possible; on the hardware infrastructure, where RAM is usually a single limiting factor for the number of search tab results open; operating system (some manual steps are easier to perform on Windows OS as system PATH can be defined directly to the spreadsheet software), number of search query iterations or modifications to be performed and depth of analysis (whether there is a need to go past the first page (1-10) results, which is defined by additional coding in XPath and Javascript).

We used a semi-automated method, consisting of data collection, assembling and mining. Semi-automation was chosen due to the need to control every single step and perform manual data quality analysis of the scraped results. Our query was designed to include two sets of parameters, where the first set was performed for cities in 2014 and another one for 2015. To enable multiple searches at once, we created automated browser container (code below), into which we pasted 507x2 (complete list of cities, identified in Google Analytics dataset, for the years 2014 and 2015).

```
<html>
<head>
<script>
function search()
{
    var queries =
document.getElementsByTagName('textarea')[0].value.split(/[\r\n]/);
    for(var i = 0, j = queries.length; i < j; i++)
        if(queries[i])
            window.open('http://www.google.com/search?q=' + queries[i]);
}
</script>
</head>
<body>
<textarea style="height:50%;width:100%"></textarea>
<input type="button" value="Start!" onclick="search()">
</body>
</html>
```

Two examples of the query performed for Bristol are presented below. Because the aim of our search was to collect information about actual floods rather than predicted or expected, we excluded any of the terms pointing to the probability of the event, e.g., 'warning', 'alert', 'risk', 'weather forecast'. We also excluded any

*Author for correspondence (Nataliya.Tkachenko@warwick.ac.uk).
†Present address: Warwick Institute for the Science of Cities, University of Warwick, Coventry, CV4 7AL, UK

climate-related events or meetings that took place in the locality during the time period of interest and might be returned with results: e.g., 'university', 'conference', and 'workshop':

```
'allintext:flooded bristol 2014 -warning -alert -risk -weather -forecast -
university –conference -workshop'
'allintext:flooded bristol 2015 -warning -alert -risk -weather -forecast -
university –conference -workshop'
```

Without getting into coding details, similar results could be obtained using the Multiple Tabs Search extension for the Google Chrome browser.

Because our analysis didn't require the complete search results, only the most relevant ones, we collected the top ten returns (the first page of the Google Results), using the Chrome Extension Data Miner with help of the following XPath rules:

**Name**	**XPath**	**Target**
Id	//div[@class='g']	Row
Link	//div[@class='rc']/h3/a	Column
URL	//div[@class='rc']/h3/a/@href	Column
BasicDescription	//div[@class='rc']/div/div/span[@class='st']	Column
Cite	//div[@class='rc']/div/div/div/cite	Column
Options	//div[@class='rc']/div/div/div/div	Column
MiscSnippet	//div[@class='rc']/div/div	Column

Another minimal coding option for extraction of the top URLs from queries that doesn't involve multiple tabs is possible using another popular Chrome extension, Linkclump. This tool requires repetitive manual collation of the highlighted search results into spreadsheet processing software, e.g., Googledocs, Excel or LibreOffice.

If the coding option is preferred, it is also possible to make use of this Python code below, which uses BeautifulSoup4. This option may be preferred due to the fact that the rest of the data analysis is performed in Python.

```
>>>import bs4, sys, requests, webbrowser
>>>res=requests.get('http://google.com/search?q=' + ''.join(sys.argv[1:]))
>>>soup=bs4.beautifulSoup(res.text)
>>>linkElems=soup.select('.r a')
>>>numOpen=min(10, len(linkElems))
>>>def save_to_file(*numOpen):
     with open('/path/to/filename.txt',mode='wt',encoding='utf-8') as myfile:
          for lines in numOpen:
              myfile.write('\n'.join(str(line) for line in lines))
              myfile.write('\n')
```

The lists of URLs collected were subsequently analysed in order to verify whether any case of flooding had been recorded in the locality during period from April 2014 to March 2015. For this, we assembled the list of dates in several formats (e.g., 'Date Month Year' or 'dd-mm-yyyy') to cover the twelve months period of the study, and performed iterative data scraping across both lists of URLs ('2014' and '2015') collected, having first extracted the text to which they pointed:

```
>>>from bs4 import BeautifulSoup
>>>twenty14=BeautifulSoup(html)
>>>twenty14_text=twenty14.get_text()
```

Finally, we iterated the list of dates over the text datasets in order to code each city according to whether or not it was flooded on each date:

```
>>>with open('/path/to/twenty14_text.txt') as f:
        twenty14=f.read()
>>>with open('/path/to/Date_Month_Year.txt') as f:
        for date in (line.strip() for line in f):
            if date in twenty14:
               print('1')
            else:
               print('0')
```

Combined results from these iterations are presented below.

**PC1|α≥0.5**		**PC2|α≥0.5**		**PC3|α≥0.5**		**PC4|α≥0.5**	
Hull	‘1’	Chesterfield	‘0’	Droitwich Spa	‘0’	Cramlington	‘0’
Bournemouth	‘1’	Durham	‘0’	Cwmbran	‘0’	Huddersfield	‘0’
Liverpool	‘0’	Towcester	‘0’	Hedge End	‘0’	Rushden	‘0’
Southampton	‘1’	Oldham	‘0’	Havant	‘0’	Clevedon	‘1’
Cambridge	‘1’	Swindon	‘0’	Brockenhurst	‘0’	Calne	‘0’
Teddington	‘0’	Ilkeston	‘1’	West Malling	‘0’	Runcorn	‘1’
Sheffield	‘1’	Rotherham	‘0’	Haywards Heath	‘0’	Bradford-on-Avon	‘0’
Northampton	‘0’	Doncaster	‘1’	Tonbridge	‘0’	Iver	‘0’
Coventry	‘1’	Sleaford	‘1’	Gravesend	‘1’	Scarborough	‘0’
Manchester	‘1’	Birchwood	‘1’	East Grinstead	‘1’	Pontefract	‘1’
Birmingham	‘1’	Derby	‘1’	Ashford	‘0’	Wigan	‘0’
Loughborough	‘0’	Kingswinford	‘1’	Chinnor	‘0’	Grantham	‘0’
Wrexham	‘1’	Long Eaton	‘1’	Attleborough	‘0’	Barnstaple	‘1’
Blackburn	‘0’	Burton-upon-Trent	‘1’	Alcester	‘0’	Tamworth	‘0’
Nottingham	‘1’	Louth	‘1’	Hailsham	‘0’	Lancaster	‘0’
Barnstaple	‘1’	Harrogate	‘0’	Hastings	‘0’	Brough	‘0’
Norwich	‘1’	Grimsby	‘1’	Geddington	‘0’	Glossop	‘0’
Leicester	‘1’	Hucknall	‘1’	Horley	‘0’	Lechlade	‘0’
Cardiff	‘1’	Sheffield	‘1’	Sandbach	‘1’	Sittingbourne	‘1’
Grimsby	‘1’	Staplehurst	‘0’	Carmarthen	‘0’	Sutton-in-Ashfield	‘0’
Doncaster	‘1’	Nottingham	‘1’	Bridgwater	‘0’	Bognor Regis	‘0’
Frome	‘1’	Wakefield	‘1’	Hereford	‘0’	Stourport-on-Severn	‘0’
Newcastle-upon-Tyne	‘0’	Grantham	‘0’	Buntingford	‘0’	Rugby	‘0’
Burton-upon-Trent	‘1’	Barnsley	‘1’	Lakenheath	‘0’	Darwen	‘0’
Kingswinford	‘1’	Brentford	‘0’	Cardiff	‘1’	March	‘1’
York	‘0’	Runcorn	‘1’	Oldbury	‘1’	Sidmouth	‘1’
Eastbourne	‘1’	Horncastle	‘0’	Royal Tunbridge Wells	‘1’	Lenham	‘0’
Long Eaton	‘1’	Bude	‘1’	Bishop’s Stortford	‘1’	Cleethorpes	‘1’
Belper	‘1’	Pontefract	‘1’	Farnham	‘0’	Horncastle	‘0’
Edwalton	‘0’	Bolton	‘1’	Abergavenny	‘0’	Staplehurst	‘0’
Beverley	‘1’	Stourport-on-Severn	‘0’	Torquay	‘0’		
Preston	‘1’	Loughborough	‘0’	Swansea	‘0’		
Clevedon	‘1’	Rugby	‘0’	Worcester	‘0’		
Bolton	‘0’	Middlesbrough	‘0’	Bath	‘0’		
Tamworth	‘0’	Bradford-on-Avon	‘0’	Maidstone	‘0’		
Gloucester	‘0’	Royal Leamington Spa	‘0’	Eastbourne	‘0’		
Widnes	‘1’	Hinckley	‘1’	Macclesfield	‘1’		
Brough	‘0’			King’s Lynn	‘0’		
Glossop	‘0’			Retford	‘1’		
Lechlade	‘0’			Brighton	‘1’		
Sittingbourne	‘1’			Carlisle	‘1’		
Sutton-in-Ashfield	‘1’						
Castleford	‘1’						
Lenham	‘1’						
Barnsley	‘1’						
Wakefield	‘1’						
Cleethorpes	‘0’						
Bradford-on-	‘0’						

Avon	
Rushden	‘1’
Scunthorpe	‘1’
Oldham	‘0’
Horncastle	‘0’
Swansea	‘1’
Stourport-on-Severn	‘0’
Swindon	‘0’
Camberley	‘0’
Bristol	‘0’
Staplehurst	‘0’
Farnborough	‘0’
Stroud	‘0’
Retford	‘1’
Leeds	‘0’
Gateshead	‘0’
Peterborough	‘0’
Scarborough	‘0’
Harrogate	‘0’
Sleaford	‘1’
Stafford	‘1’
Brentford	‘1’
Wigan	‘0’
March	‘1’
Grantham	‘0’
Evesham	‘0’
Worthing	‘1’
Towcester	‘0’
Lancaster	‘0’
Luton	‘0’
Worcester	‘0’
Cheltenham	‘0’
Huntingdon	‘0’
Bishop’s Cleeve	‘0’
Brighton	‘1’
Runcorn	‘1’
Durham	‘0’
Kenilworth	‘0’
Sidmouth	‘1’
Pontefract	‘1’
Ilkeston	‘1’
Louth	‘1’
Hertford	‘0’
Ipswich	‘0’
Lymington	‘0’
Newport	‘0’
Warrington	‘0’
Milton Keynes	‘1’
Huddersfield	‘0’
